# Pseudohyperphosphatemia in Multiple Myeloma: A Systematic Review of Case Reports and Case Series

**DOI:** 10.1002/jcla.70281

**Published:** 2026-06-10

**Authors:** Mahsa Dabir, Maryam Kamrani Mousavi, Mohana Mohamadi, Ali Maleki

**Affiliations:** ^1^ Department of Medical Laboratory Science, School of Paramedical Kermanshah University of Medical Sciences Kermanshah Iran; ^2^ Student Research Committee Kermanshah University of Medical Sciences Kermanshah Iran

**Keywords:** hyperphosphatemia, monoclonal gammopathy, multiple myeloma, phosphate assay interference, Pseudohyperphosphatemia

## Abstract

**Background:**

Pseudohyperphosphatemia (PHP) is a laboratory artifact that may occur in patients with multiple myeloma (MM) due to interference from circulating paraproteins in colorimetric phosphate assays. Misinterpretation of this artifact may lead to unnecessary diagnostic evaluation or inappropriate treatment. This systematic review aims to investigate the reported cases of PHP in patients with MM to describe the features used to distinguish it from true hyperphosphatemia.

**Methods:**

A comprehensive search of PubMed, Scopus, and Web of Science was conducted in November 2025 to identify English‐language case reports and series involving MM patients with hyperphosphatemia. Studies were included if they reported hyperphosphatemia or pseudohyperphosphatemia in patients with multiple myeloma. Data extraction and quality assessment were performed independently by two reviewers using JBI tools.

**Results:**

Fourteen studies encompassing 19 patients were included. IgG‐kappa was the most frequent paraprotein subtype (*n* = 16). Most patients exhibited elevated phosphate levels via phosphomolybdate‐based assays, which normalized with protein precipitation, dilutional testing, or alternative methods such as atomic emission spectroscopy. Only one case of true hyperphosphatemia was confirmed in the setting of renal dysfunction. Several reports described decreases in measured phosphate levels following chemotherapy and reduction of paraprotein concentrations; however, the available data were limited and insufficient to establish a formal correlation.

**Conclusion:**

Reported cases of hyperphosphatemia in patients with MM are often attributable to PHP caused by paraprotein interference. Further studies are required to better characterize the mechanisms and clinical implications of this laboratory interference.

## Graphical abstract

Pseudohyperphosphatemia (PHP) is a clinically significant laboratory artifact in patients with multiple myeloma (MM). It is often caused by paraprotein interference with phosphate assays. This systematic review summarizes reported cases, underlying mechanisms, and diagnostic approaches to distinguish spurious from true hyperphosphatemia. Awareness of this phenomenon can prevent unnecessary investigations and inappropriate treatment.

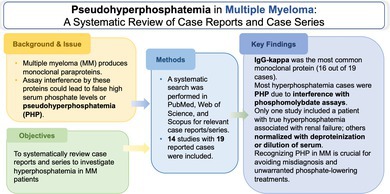

## 1 Introduction

Multiple myeloma is an incurable hematological malignancy characterized by uncontrolled growth of monoclonal plasma cells in the bone marrow that leads to the overproduction of immunoglobulin chains [1]. Paraproteins are abnormal immunoglobulins produced in excess by malignant plasma cells in multiple myeloma (MM). These proteins can be of various immunoglobulin subtypes, with IgG being the most common, followed by IgA. The light chain component is often kappa, though lambda chains are also reported [2]. MM can lead to a range of biochemical abnormalities [3]. Among these, abnormalities in serum phosphate measurements have occasionally been reported in patients with MM [4]. Serum phosphate levels are regulated by a complex interaction between intestinal absorption, renal tubular reabsorption, and the transcellular movement of phosphate between intracellular fluid and bone storage pools [5]. Renal disorders result in phosphate imbalance in the body, leading to hyperphosphatemia, a condition that can lead to serious, life‐threatening outcomes, including bone mineral imbalances and vascular calcification [6]. In patients with MM, hyperphosphatemia is an uncommon finding unless there is significant renal impairment [7].

Paraproteins can exert a wide range of effects on laboratory assays and clinical biochemistry. In MM patients, this interference leads to falsely elevated phosphate measurements, a phenomenon known as pseudohyperphosphatemia (PHP) [8]. Most cases of PHP in MM are associated with the IgG subtype [9]. The interference typically arises from the interaction between paraproteins and reagents used in standard phosphomolybdate‐based assays. In the standard phosphomolybdate assays, inorganic phosphate reacts with ammonium molybdate in an acidic medium to form a phosphomolybdate complex that is measured spectrophotometrically [10, 11]. High concentrations of paraproteins could precipitate or increase turbidity in the acidic reaction mixture. This phenomenon produces nonspecific absorbance that is interpreted as elevated phosphate levels [10]. Alternative methods, such as pretreatment with sulfosalicylic acid, trichloroacetic acid, or perchloric acid, as well as using dry chemistry analyzers with barium sulfate filtration or atomic emission spectroscopy, can eliminate this interference and yield accurate phosphate measurements [12]. Chemotherapy regimens that reduce paraprotein levels (e.g., melphalan, prednisone, doxorubicin, cyclophosphamide) also lead to normalization of pseudohyperphosphatemia. Recognition of pseudohyperphosphatemia is crucial to avoid unnecessary interventions, such as unwarranted phosphate‐lowering strategies or stopping essential medications [7].

The current study is the first systematic review investigating reported cases of PHP in MM patients.

## 2 Materials and Methods

This systematic review was conducted in adherence with Preferred Reporting Items for Systematic Reviews and Meta‐Analyses (PRISMA) guidelines [13, 14].

### 2.1 Eligibility Criteria

Studies were considered eligible for inclusion if they reported on patients diagnosed with multiple myeloma, including different immunoglobulin isotypes such as IgG, IgA, and light‐chain types (kappa or lambda). Historical terminology and diagnostic variants used in earlier literature, such as myelomatosis or Kahler's disease, were also considered eligible when clearly referring to MM. Reports describing plasmacytoma were included only when PHP associated with paraproteinemia was described. Only case reports and case series published in English were included. No restrictions were applied regarding publication date to ensure the capture of all relevant literature. Studies were excluded if they were reviews, editorials, conference abstracts lacking full clinical data, animal studies, duplicate reports, or reports that did not clearly document a diagnosis of multiple myeloma and a phosphate abnormality sufficient to evaluate possible pseudohyperphosphatemia.

### 2.2 Search Strategy

A systematic literature search was conducted in November 2025 using three major electronic databases: PubMed, Scopus, and Web of Science. The search was designed to identify case reports and case series related to hyperphosphatemia and variants of PHP in patients with MM or other plasma cell disorders. Search terms combined free‐text keywords related to PHP and plasma cell neoplasms. The primary terms included variations of “hyperphosphatemia”, “pseudohyperphosphatemia,” “spurious hyperphosphatemia,” and diagnostic descriptors such as “myeloma,” “plasma cell,” “plasma cells,” “plasmacell,” “plasmacytoma,” “myelomatosis,” “Kahler's disease,” and “Kahler disease.” This strategy yielded 44 records in PubMed, 58 in Web of Science, and 150 in Scopus. All retrieved records were screened for eligibility based on predefined criteria. The complete search strings for each database are provided in the Table S1.

### 2.3 Study Selection Process

After duplicate removal using EndNote, the titles and abstracts of all retrieved records were independently screened by two reviewers. Full texts were obtained for all potentially eligible articles or those with unclear eligibility. Disagreements were resolved by discussion or consultation with a third reviewer.

### 2.4 Data Analysis

Descriptive approaches were employed to summarize the extracted data. Extracted variables included patient demographics, immunoglobulin subtype, reported serum phosphate levels, assay methods used for phosphate measurement, and approaches used to correct assay interference. Where sufficient data were available, simple descriptive statistics were calculated, including counts, proportions, and median phosphate levels with ranges. No formal statistical analyses were performed since the available evidence consisted primarily of case reports and small case series. Observations regarding changes in phosphate measurements following reduction of paraprotein levels were described narratively.

### 2.5 Data Extraction and Quality Assessment

Two independent reviewers extracted data from each of the 14 included studies using a standardized form. Extracted variables included the phosphate measurement method or assay, use, and type of deproteinization technique (if reported), the analytical platform or analyzer used, measured serum phosphate levels before and after correction, and the corresponding reference intervals. For case reports and case series, the Joanna Briggs Institute (JBI) critical appraisal checklists were employed [15]. Quality assessment focused on potential biases such as selection bias, measurement bias related to phosphate assays, and reporting bias.

## 3 Results

**FIGURE 1:**
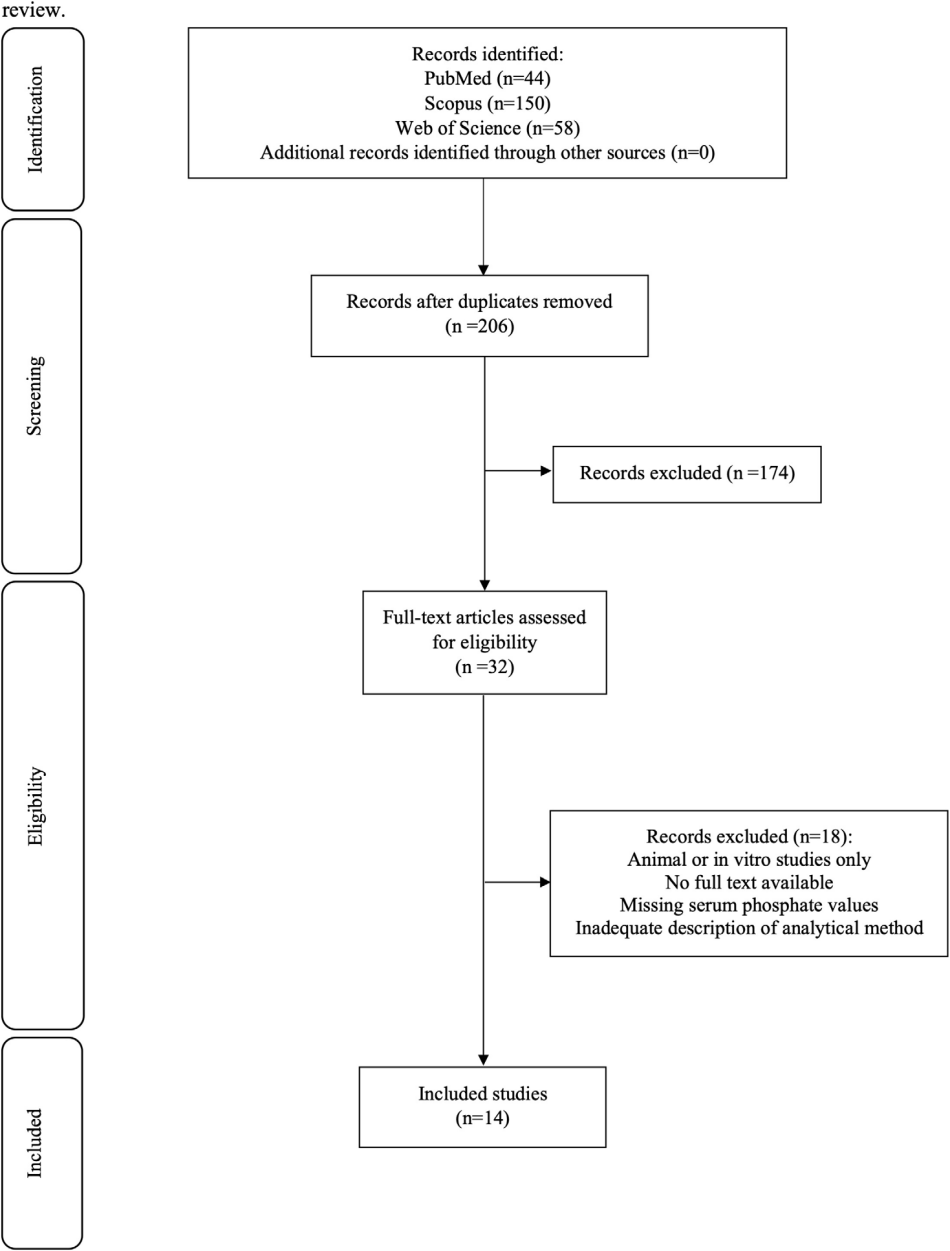
PRISMA 2020 flow diagram showing the process of study selection for the systematic review.

A total of 249 records were identified through database searches, including 44 from PubMed, 150 from Scopus, and 58 from Web of Science. After removal of 43 duplicate entries, 206 records remained for title and abstract screening. Of these, 174 were excluded based on irrelevance or failure to meet the predefined eligibility criteria. The full texts of 32 potentially relevant articles were then reviewed in detail, resulting in the inclusion of 14 studies in the final analysis. The complete study selection process is illustrated in the PRISMA 2020 flow diagram (Figure 1).

### 3.1 Study Characteristics

Cases originated from various countries, including the USA (*n* = 2), Israel (*n* = 2), and Turkey (*n* = 2). The rest (*n* = 8) were from various countries, including England, Tunisia, Germany, Thailand, South Korea, New Zealand, India, and France. Among the 19 reported patients, IgG‐kappa was the most frequently described paraprotein subtype (16/19, 84.2%), followed by IgG‐lambda (2/19, 10.5%); one report did not clearly specify the light chain subtype. Out of the 14 studies with available phosphate assay data, 13 demonstrated lower phosphate levels after switching to a different measurement assay.

### 3.2 Summary of Clinical Findings

Across the pooled cases, phosphate values before correction were elevated. Measurement units and reporting formats varied across studies and limited direct quantitative comparison. Notably, only one patient presented with an underlying renal disease and an elevated phosphate level (8.6 mmol/L; reference interval [RI]: 0.68–1.68 mmol/L). Serum samples underwent five‐ and ten‐fold dilution and were re‐assessed, this time revealing a mildly elevated serum phosphate (1.82 mmol/L; RI: 0.68–1.68 mmol/L). The authors concluded that this slight elevation was due to the underlying chronic renal disease [16].

The monoclonal component of serum was elevated in all patients across the included studies. IgG was the only reported heavy chain subtype among reported patients (*n* = 19). Kappa was the most commonly reported light chain (*n* = 16), followed by Lambda (*n* = 2), in patients with available data. McCloskey et al. described a 40‐year‐old woman with IgG‐lambda multiple myeloma who presented with an elevated serum phosphate level of 2.36 mmol/L (RI: 0.8–1.4 mmol/L) in the absence of any clinical signs of hyperphosphatemia [17]. Similarly, Sonnenblick et al. reported a case of IgG‐lambda MM with elevated serum phosphate levels accompanied by normal serum calcium (8.9 mg/dL) and creatinine (11 mg/L) levels [18]. In this set of reported cases, IgG‐kappa was the most frequently described combination among those with available data.

### 3.3 Assay‐Based Findings

Initial phosphate measurements were often acquired from phosphomolybdate‐based assays, which are prone to interference from paraproteins. For instance, Cheikhrouhou et al. reported an initial serum phosphate level of 3.61 mmol/L (RI: 0.8–1.35 mmol/L), which normalized to 1.1 mmol/L after chemotherapy [19].

Latus et al. reported levels as high as 8.6 mmol/L (RI: 0.68–1.68 mmol/L), which decreased to near‐normal (1.82 mmol/L) upon dilutional testing [16]. Chemical deproteinization methods were also used to eliminate assay interference. Treatment with sulfosalicylic acid (SSA), trichloroacetic acid, and nitric acid effectively normalized initially elevated phosphate levels. Barutçuoglu et al. demonstrated a decline from 6.46–13.14 mmol/L (phosphomolybdate assay) to 0.36–1.84 mmol/L following SSA pretreatment [9]. Alternative analytical platforms that avoided protein interference also produced lower phosphate measurements. Atomic emission spectroscopy demonstrated normal phosphate levels (1.92–2.01 mmol/L; RI: 1.62–2.40 mmol/L) in contrast to elevated readings from methods that did not filter serum proteins like the RA1000 and Technicon SMAC analyzers [9]. Alternative dry chemistry platforms, such as the Ortho Vitros and multilayered film technology analyzers with barium sulfate filtration, also provided phosphate measurements not affected by paraprotein interference. Chakraborty et al. reported a drop in serum phosphate from 7.75 mmol/L, measured by the Roche Cobas system, to 1.32 mmol/L when reassessed using a barium sulfate‐layered analyzer [20]. Enzymatic and vanadate‐based assays were the other measurement assays utilized [18]. Hawkins et al. reported that sodium chloride‐containing reagents were linked to artifactual precipitates and aberrant absorbance patterns, whereas NaCl‐free assays or acid‐pretreated samples eliminated these interferences [21]. Details regarding measurement assays are listed in Table 1.

**TABLE 1:** Analytical and clinical characteristics of included studies in the systematic review of pseudohyperphosphatemia in multiple myeloma.

Ref.	Assay/method details	Deproteinization method	Analyzer	Phosphate levels (mmol/L)	Reference interval (mmol/L)
(1)	Phosphomolybdate assay	NP	NR	Initial evaluation: 3.61 Following chemotherapy: 1.1	0.8–1.35
		Performed deproteinization but did not specify.	NR	1	0.8–1.35
(2)	A wet chemistry phosphomolybdate assay with UV absorbance at 340 nm	NP	Roche Cobas	Prior to admission: 0.63–1.53 On admission: 1.71	0.26–0.47
		1:5 dilution	Roche Cobas	Within normal range	0.26–0.47
	A dry chemistry phosphomolybdate assay, in which a reduced molybdenum blue complex was formed and measured by absorbance at 670–680 nm.	Includes built‐in protein filtration	Ortho Vitros	Prior to admission: Within normal range	NR
		1:5 dilution	Ortho Vitros	Within normal range	NR
(3)	A Chromogenic colorimetric assay, likely phosphomolybdate‐based	NP	Hitachi 737	2.36	0.8–1.4
	Chromogenic assay	NP	RA1000	2.49–2.73	0.7–1.5
	Chromogenic assay	Performed deproteinization but did not specify.	Technicon SMAC	1.46–1.53	0.7–1.5
	Measures total inorganic and organic phosphate via elemental detection	NA	AES	1.92–2.01	1.62–2.40
(4)	Standard phosphomolybdate assay	NP	NR	2.32–4.75	0.77–1.45
	Sepharose bead method with anti‐human IgG and colorimetric assay	IgG was removed via the anti‐IgG beads	NR	2.58 in the diluted serum sample 1.61 after serum IgG removal	NR
(5)	Standard phosphomolybdate assay	NP	NR	8.6	0.68–1.68
		1:5 and 1:10 dilution	NR	1.82	0.68–1.68
(6)	Phosphomolybdate assay, with absorbance measured at 340 nm	NP	Cobas 8000 (Roche Diagnostics)	5.17–7.75	0.81–1.45
		SSA	Cobas 8000	Within normal range	0.81–1.45
		Sample dilution	Cobas 8000	Within normal range	0.81–1.45
(7)	Phosphomolybdate assay, with absorbance measured at 340 nm	NP	NR	Initial evaluation: 4.11 After the 3rd cycle of chemotherapy: 4.23 Same sample, before deproteinization: 3.39	NR
		20% SSA	NR	1.13	NR
(8)	The phosphomolybdate assay, with absorbance measured at 340 nm and 376 nm, contains NaCl.	NP	Hitachi 704	P1: 6.60 P2: 2.55	0.70–1.50
	Phosphomolybdate assay with absorbance measured at 340 nm	NP	CHEM I	P1: 1.70 P2: 1.60	0.70–1.50
			RA1000	P1: 1.52 P2: 1.43	0.70–1.50
	Phosphomolybdate assay with absorbance measured at 340 nm and 380 nm	NP	Abbott EPX	P1: 1.38 P2:1.54	0.70–1.50
(9)	A wet chemistry phosphomolybdate assay	NP	Beckman AU480	7.75	0.81–1.45
		1:2 dilution	Beckman AU480	3.07	0.81–1.45
		1:4 dilution	Beckman AU480	2.91	0.81–1.45
		1:8 dilution	Beckman AU480	0.16	0.81–1.45
		1:16 dilution	Beckman AU480	0.10	0.81–1.45
		NP	Roche platform	Similar to the initial level, not numerically reported	NR
	Multilayer film technology	Barium sulfate filter layer	Vitros 250, Ortho Clinical Diagnostics	1.32	0.74–1.45
(10)	A modified phosphomolybdate assay based on the reaction of phosphate with ammonium molybdate, without reduction.	NP	Technicon Dax‐48	6.46–13.14	0.74–1.45
		SSA	Technicon Dax‐48	0.36–1.84	0.74–1.45
(11)	Phosphomolybdate assay, based on the Fiske‐Subbarow 1925 method	NP	NR	Initial evaluation: 10.2 Following chemotherapy: 1	0.8–1.4
		Performed deproteinization but did not specify.	NR	1.7	0.8–1.4
(12)	Standard phosphomolybdate assay	NP	NR	Initial evaluation: 6.46 Pre‐TPE: 5.62 Following TPE: 1.16 months later: 1.16–1.55	NR
		SSA	NR	Within normal range	NR
(13)	Phosphomolybdate assay with absorbance measured at 690 nm	NP	NR	On admission P1: 5.17–5.49 P2: 2.46 P3: 3.20–3.97	0.90–1.45
				During hospitalization P1: NR P2: 6.33–7.30 P3: NR	0.90–1.45
				Following treatment: P1: NR P2: NR P3: 2.04 (later elevated to 4.23 due to relapse)	0.90–1.45
(14)	Phosphomolybdate assay with absorbance measured at 690 nm, using Ferrous sulfate as the reducing agent	NP	Discrete analyzer Model 203‐S	P1: 2.84 P2: 2.53 P3: 1.37	0.26–0.44
	Vanadate method for phosphate measurement, absorbance measured at 420 nm	A dialysis step	AutoAnalyzer II, Technicon Instruments Corp.	P1: 0.51 P2: 0.36 P3: 0.41	0.21–0.45
	ICP atomic emission spectroscopy	Nitric and perchloric acids	Plasmatherm RF Generator Model JY 48	P1: 0.65 P2: 0.61 P3: 0.63	0.58–0.68

*Note:* Unit conversion was performed using the molecular weight of inorganic phosphate (1 mg/dL = 0.323 mmol/L). Values stated as “within normal range” were not quantitatively reported in the full text of the relative paper. Units have been converted from mg/dL and mg/L to mmol/L via standard conversion factors.

Abbreviations: AES, Atomic Emission Spectroscopy; NA, not applicable; NP, not performed; NR, not reported; P, patient; Ref,reference; SSA, salicylic acid; TCA, Trichloroacetic acid; TPE, therapeutic plasma exchange.

#### 3.3.1 Treatment Approaches

Treatment‐related information was reported in only a subset of cases. In the available reports, management primarily involved treatment of the underlying multiple myeloma rather than specific therapy for true hyperphosphatemia. Cheikhrouhou et al. documented a decline in serum phosphate from 3.61 to 1.1 mmol/L (RI: 0.8–1.35 mmol/L) following six cycles of chemotherapy with melphalan and prednisone. This reduction occurred in parallel with a decrease in the monoclonal component [19]. In contrast, Lee et al. reported a patient treated for three months with melphalan, prednisolone, and calcium carbonate (CaCO_3_), who showed no reduction in serum phosphate, which remained elevated at 4.23 mmol/L, despite a decrease in total serum protein (from 11.3 to 9.9 g/dL) and IgG levels (from 9810 to 6250 mg/dL). Following the diagnosis of pseudohyperphosphatemia, calcium carbonate therapy was discontinued for the patient [7].

McCloskey et al. described a patient with IgG‐kappa MM and pseudohyperphosphatemia who received a regimen of doxorubicin, carmustine, cyclophosphamide, melphalan, and prednisolone, along with a placebo of clodronate [17]. After one month, serum IgG dropped from 72 g/L to 33 g/L, paralleled by a decrease in serum phosphate from 2.08 to 1.64 mmol/L. However, after replacing clodronate with cyclophosphamide, serum globulin levels increased, which was again accompanied by a rise in phosphate levels (2.56 mmol/L).

## 4 Discussion

This review provides a structured description of reported cases of PHP in MM. The patterns observed are consistent with earlier reports of assay interference by monoclonal proteins, although the limited evidence base precludes firm conclusions.

One of the most prominent features emerging from our review is an apparent predominance of IgG‐kappa among reported PHP cases. Previous studies, such as those by McCloskey et al. and Sonnenblick et al., described individual cases of PHP involving various immunoglobulin isotypes, including IgG‐lambda [17, 18]. In contrast, in this small set of published cases, 16 of 18 involved IgG‐kappa, although this pattern may reflect publication or reporting biases rather than a true biological predilection. This consistency raises the question of whether IgG‐kappa has unique physicochemical properties, such as molecular size, solubility, or interaction with assay reagents, that make it more prone to causing false phosphate elevations. Alternatively, the apparent predominance may reflect reporting bias, where more unusual or extreme cases involving IgG‐kappa are more likely to be published. Regardless, this pattern warrants further mechanistic exploration [22].

Compared to earlier literature, which primarily reported anecdotal observations or isolated case reports, our review integrates findings from multiple geographical regions and assay platforms, allowing a broader assessment of PHP as a systemic analytical challenge. For example, while Barutçuoglu et al. and Chakraborty et al. each presented single‐case demonstrations of normalization using sulfosalicylic acid (SSA) or dry chemistry methods, our review is consistent with previous observations [9, 20]. In particular, the effectiveness of protein precipitation and dilutional testing (e.g., 1:10 or 1:16) was consistently observed, suggesting that these methods may be useful in cases where PHP is suspected, although prospective validation is needed before routine adoption.

A point of significant clinical interest is the connection between serum phosphate levels and paraprotein burden. Previous reports noted resolution of PHP following chemotherapy but provided only limited data [17, 19]. In our review, multiple cases demonstrated phosphate normalization in parallel with decreases in IgG or total protein and recurrence of pseudohyperphosphatemia with disease relapse. This dynamic pattern, previously underemphasized, suggests that phosphate elevation, if unexplained by renal dysfunction or other causes, may reflect disease activity in some cases, although this observation requires prospective evaluation. This potential application, however, requires cautious interpretation and prospective validation, as standard assays are highly variable in sensitivity and specificity [23]. Our observations were drawn from a very small number of heterogeneous case reports, while most studies did not provide standardized longitudinal paired measurements of phosphate and paraprotein burden. No formal correlation can be established from the available evidence, and this finding should be interpreted as hypothesis‐generating rather than confirmatory. Future studies with serial phosphate measurements paired with serum protein electrophoresis, immunofixation, or free light chain data are needed to assess this relationship more rigorously.

From an analytical standpoint, our findings highlight significant vulnerabilities in commonly used phosphate assays. Colorimetric phosphomolybdate‐based methods remain the most widely used in routine clinical laboratories, yet our synthesis reinforces their susceptibility to paraprotein interference [24]. While Hawkins had hypothesized that sodium chloride‐containing buffers contributed to assay artifacts, this hypothesis had remained largely theoretical [21]. Our inclusion of cases that used NaCl‐free reagents or acid pretreatment protocols suggests that these modifications can eliminate false elevations. This has practical implications: Laboratories using standard reagents without accounting for protein interference may inadvertently report misleading phosphate results in MM patients. Moreover, platforms such as atomic emission spectroscopy (AES) and vanadate‐based assays, which consistently produced normal phosphate levels in our review, should be considered as confirmatory tools when PHP is suspected.

Importantly, this analytical variability has direct clinical consequences. As shown in our analysis and previously highlighted by Lee et al., misdiagnosis of PHP has led to unnecessary treatment interventions, including phosphate binders like calcium carbonate and inappropriate diagnostic investigations [7]. In some cases, this may have contributed to delays in the proper treatment of the underlying myeloma. The broader documentation of such instances across multiple centers in our review suggests that this is not a sporadic issue, but may represent a recurrent challenge in laboratory‐clinical communication, resulting in unnecessary treatment and delayed diagnosis [7, 17, 19]. Therefore, a high index of suspicion, especially in asymptomatic patients with high phosphate levels and preserved renal function, is essential to avoid patient harm.

Despite the insights gained, several limitations must be acknowledged. Most of the included studies were case reports or small series, which inherently limit generalizability. The lack of standardized diagnostic criteria or interference thresholds for PHP prevents definitive conclusions about prevalence and risk stratification. Furthermore, assay heterogeneity across institutions complicates direct comparisons. There may also be publication bias, as cases of mild or undetected PHP may remain unpublished, skewing the observed distribution toward more dramatic instances. Lastly, the absence of detailed biochemical or structural analyses of the interfering paraproteins limits mechanistic understanding. Because the review was based entirely on case reports and small case series without a control group, causality and the strength of any observed associations cannot be established.

Given these limitations, several directions for future research emerge. Prospective cohort studies should evaluate the frequency and predictors of PHP among unselected MM populations. Detailed biochemical characterization of different immunoglobulin subtypes and their reactivity with various assay reagents may shed light on the mechanisms of interference. Additionally, cost‐effectiveness analyses are needed to assess whether incorporating dilutional or alternative confirmatory testing into routine workflows is justified in terms of reducing misdiagnosis and inappropriate treatment.

In conclusion, our review reaffirms PHP as a frequent and clinically significant laboratory artifact in patients with IgG‐kappa multiple myeloma. By consolidating evidence from 14 studies, we provide a more structured understanding of its pathophysiology, analytical pitfalls, and clinical implications. Recognition of this phenomenon is critical to preventing misinterpretation of laboratory results, avoiding unnecessary interventions, and improving overall patient management. Greater collaboration between clinical teams and laboratory medicine is essential to ensure appropriate diagnostic pathways are followed when unexplained hyperphosphatemia is encountered in this patient population.

## Funding

This review received no external funding.

## Ethics Statement

The authors have nothing to report.

## Consent

The authors have nothing to report.

## Conflicts of Interest

The authors declare no conflicts of interest.

## Supporting information


**Table S1:** Complete search strategy with database‐specific terms.

## Data Availability

The data that support the findings of this study are available from the corresponding author upon reasonable request.
