# Supplementary material for: Pseudohyperphosphatemia in Multiple Myeloma: A Systematic Review of Case Reports and Case Series
Source: J Clin Lab Anal. 2026 Jun 10;40(15):e70281. doi: 10.1002/jcla.70281 (PMC13399738; doi:10.1002/jcla.70281)
Supplement: Supplementary file 1 [file JCLA-40-e70281-s001.docx]

**Supplementary** **Table 1**. Complete search strategy with database-specific terms.

| terms | |
| --- | --- |
| "Hyperphosphatemia" OR "Hyperphosphatemias" OR “pseudohyperphosphatemia” OR “spurious hyperphosphatemia” OR “factitious hyperphosphatemia” | “myeloma” OR “multiple myeloma” OR “plasma cell” OR “plasma cells” OR plasmacell OR plasmacytoma OR myelomatosis OR “Kahler’s disease” OR “Kahler disease” |

November, 2025

| PubMed | #1 AND #2 | 44 |
| --- | --- | --- |
| Scopus | #1 AND #2 | 150 |
| Web of Science | #1 AND #2 | 58 |
| All |  | 249 |
| Duplicates |  | 43 |
